# The longitudinal BMI pattern and body composition of patients with anorexia nervosa who require urgent hospitalization: A case control study

**DOI:** 10.1186/1751-0759-5-14

**Published:** 2011-12-05

**Authors:** Keisuke Kawai, Sakino Yamashita, Takeharu Yamanaka, Motoharu Gondo, Chihiro Morita, Takehiro Nozaki, Shu Takakura, Tomokazu Hata, Yu Yamada, Sunao Matsubayashi, Masato Takii, Chiharu Kubo, Nobuyuki Sudo

**Affiliations:** 1 Department of Psychosomatic Medicine, Graduate School of Medical Science, Kyushu University, Fukuoka, Japan. 3-1-1 Higashi-ku, Fukuoka, Japan 812-8582; 2 Institute for Clinical Research, National Kyushu Cancer Center, Fukuoka, Japan; 3 Fukuoka Tokushukai Medical Center, Fukuoka, Japan

## Abstract

**Background:**

The prevention of serious physical complications in anorexia nervosa (AN) patients is important. The purpose of this study is to clarify which physical and social factors are related to the necessity for urgent hospitalization of anorexia nervosa (AN) patients in a long-term starvation state. We hypothesized that the change of longitudinal BMI, body composition and social background would be useful as an index of the necessity for urgent hospitalization.

**Methods:**

AN patients were classified into; urgent hospitalization, due to disturbance of consciousness or difficulty walking(n = 17); planned admission (n = 96); and outpatient treatment only groups (n = 136). The longitudinal BMI pattern and the clinical features of these groups were examined. In the hospitalization groups, comparison was done of body composition variation and the social background, including the educational level and advice from family members.

**Results:**

After adjusting for age and duration of illness, the BMI of the urgent hospitalization group was lower than that of the other groups at one year before hospitalization (P < 0.01) and decreased more rapidly (P < 0.01). Urgent hospitalization was associated with the fat free mass (FFM) (P < 0.01). Between the groups, no considerable difference in social factors was found.

**Conclusions:**

The longitudinal pattern of BMI and FFM may be useful for understanding the severity in AN from the viewpoint of failure of the homeostasis system.

## Introduction

Anorexia nervosa (AN) is a severe mental health disorder that is thought to be of psychogenic origins and that often results in an extreme starvation state. Serious medical complications have been reported, including electrolyte disorders, severe bone loss, and cardiac dysfunction [1,2]. Most pathophysiological complications of AN are reversible with improved nutritional status, however some physical consequences can be life-threatening [3-5].

In AN, various factors have been suggested to be responsible for the development of severe physical complications; for example, low nutritional status, drug and alcohol use, bulimia, depressed body temperature, hypotension, and electrolyte abnormality [3,5-9].

The prevention of serious physical complications by AN is important. Many practice guidelines indicate how to assess medical risk [7-10]. Judgment is not difficult on the need for urgent admission on the day of consultation using these criteria. However, the decision on whether or not urgent hospitalization will be necessary in the near future has been left to the experience of the clinician.

A longitudinal study that addresses all the pertinent criteria for outpatient and untreated patients is clinically difficult to perform. Therefore, we focused on BMI as a longitudinal factor and variables in the social background that could be objectively investigated. In addition, we assessed the body composition of AN patients who required urgent hospitalization or who had a planned hospitalization to further clarify the factors associated with urgent hospitalization. Both the physical severity and socio-demographic variables, including educational level and the advice of family members seem to be associated with a decision for hospitalization. In this paper, for the purpose of making the clinical condition apparent, we compared the pre-hospital social background and the body composition of the patients at admission.

We hypothesized that changes in the longitudinal BMI, body composition and social background would be useful as an index of this phenomenon.

## Methods

The present study was based on the principles of the Declaration of Helsinki and was approved by the Kyushu University Research and Ethics Committee. All participants provided written informed consent.

### Design

The study group was composed of 291 consecutive new outpatients with AN at the Department of Psychosomatic Medicine of Kyushu University Hospital from 2002-2008. All were diagnosed with AN according to the criteria of the Diagnostic Statistical Manual of Mental Disorders IV (DSM-IV)[11].

### Study 1

We investigated the longitudinal Body Mass Index (BMI) and clinical factors related to the necessity for urgent hospitalization of AN patients.

A study investigator interviewed all participants to determine illness duration, medical history, menstrual history, purging history, and exercise history. We calculated BMI on the first consultation day, one month before, three months before, six months before, and one year before consultation at this hospital, (5 points in total). The past BMI was determined through a letter of introduction from the previous medical institution, direct telephone inquiry to any medical institution that performed medical examinations in the past, and statements of patients and their families. BMI (kg/m^2^) was calculated as the ratio of body weight (kg) to height (m) squared. All BMI calculations were done with at least a two week interval. Patients were excluded from study if data was not available for at least three of the five BMI measurement points.

**Figure 1:**
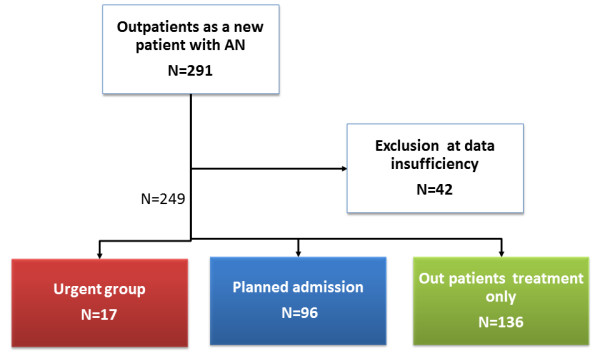
**Algorithm for classification into three groups**. Urgent group: This group consisted of patients hospitalized with disturbance of consciousness and/or difficulty walking on the day of consultation. Planned admission group: This group consisted of AN patients hospitalized for therapeutic purposes up to one year after the day of consultation. Outpatient treatment only group: Patients not admitted in the year after the initial consultation.

Patients were classified into the following three groups by differences in physical condition (Figure 1).

### Urgent group

The urgent group consisted of patients hospitalized with disturbances of consciousness and/or difficulty walking on the day of consultation.

These symptoms are absolute indicators of hospitalization[7-9]. For patients referred from other hospitals and patients with a history of urgent hospitalization, we defined a hospital day due to disturbance of consciousness or difficulty walking as the hospitalization day of this study. All causes of difficulty walking, including muscle weakness, heart failure, and infection, were included.

### Planned admission group

This group consisted of patients hospitalized for AN up to one year after the day of consultation. For patients whose BMI was not expected to increase and/or eating behavior abnormality was not expected to improve even with treatment for two months on an outpatient basis, we proposed inpatient treatment with our cognitive behavior therapy (CBT) program for eating disorders. We defined a planned admission day as the hospitalization day. In addition, urgent admission due to low weight, bradycardia, liver damage, or other physical causes was included in the planned admission group in order to eliminate the possibility of vagueness of the research data of this study. This is because the decision on the physical state of these patients on admission involves subjective factors related to the judgment of the chief physician, in contrast to disturbance of consciousness or difficulty walking as was used for the urgent group.

### Outpatient treatment only group

This group of patients was not admitted within the year after their initial consultation. Included in this group are patients who were recommended to the outpatient clinic, patients who had interrupted treatment at the outpatient clinic, patients who did not agree to proposed inpatient treatment, and patients hospitalized for a short period of several days for self- mutilation or suicide attempts, such as by wrist cutting or drug overdose.

### All control group

Planned admission group and outpatient treatment only group.

### Matched control group

A group with age and duration of disease matching the urgent hospitalization group was extracted from the all control group.

Because many factors had a serious influence on the decision for urgent hospitalization, this study was done as a case control study.

BMI change of each group was followed over one year(12, 6, 3, 1,and 0 months before). In addition, we investigated the association between BMI at the initial consultation and the necessity of urgent hospitalization.

### Study 2

Differences in the body composition and socio-demographic variables of the urgent and planned admission groups were examined. Body composition was measured after two weeks of hospitalization. Weight, fat mass(FM), lean body mass, and bone mineral content were determined for the whole body using dual-energy X-ray absorptiometry (DXA) with a HOLOGIC QDR-4500 densitometer (Hologic, Waltham, MA). All measurements were performed by an experienced technician. Fat free mass (FFM) was defined as lean body mass and bone mineral content. We investigated the association between FM and FFM and the necessity of urgent hospitalization.

Socio-demographic variables consisted of age, marital status, divorce of the parents, educational level, and employment status. Lifestyle-related factors considered were smoking and alcohol consumption. These factors were included in between group comparisons.

### Statistical Analysis

SPSS Ver. 14.0 J was used for all statistical tests. Results were presented as means ± standard deviation (SD). *P *values <0.05 were considered statistically significant. Significance at baseline was determined by the appropriate test, either chi-square test or paired f-test. We examined the change of BMI at five time-points. Comparison of between group longitudinal changes of the BMI were analyzed by repeated measures analysis of variance (ANOVA) for matched pairs. We evaluated the association between the necessity of urgent hospitalization and BMI, FM and FFM at admission using the Cochran-Armitage test of trends.

## Results

Of 291consecutive patients, 42 were excluded for lack of BMI data, leaving the data of 249 available for analysis. The treatment of 17 patients was interrupted after one consultation. The urgent group consisted of 17(7%) of the studied patients, the planned admission group 96(39%), and the group treated as outpatients only 136 (55%) (Figure 1).

### Study 1

#### Group Characteristics

##### Urgent group(n = 17)

13 of the 17 patients showed disturbance of consciousness. For 9 of the 13 patients with disturbed consciousness, hypoglycemia (<50 mg/dl) was confirmed in the emergency room. Of these, severe hypoglycemia (<30 mg/dl) was confirmed in six. 10 of the 17 patients showed difficulty in walking. Five patients without disturbance of consciousness were urgently hospitalized only because of difficulty in walking, one had infective endocarditis, and another dehydration due to acute enteritis. Four patients had low body temperature (<35 degrees). In addition, one patient had a severe infection.

##### Planned admission group(n = 96)

80 of the 96 patients were admitted for therapeutic purposes. 16 patients were urgently hospitalized on a consultation day or the following day with associated physical factors such as bradycardia, severe liver damage, electrolyte abnormality, or low body weight. These patients had set a date to be hospitalized on the initial diagnosis day and were hospitalized within a few days after consultation.

##### Outpatient treatment only group (n = 136)

17 patients from this group interrupted treatment after one consultation.

**Figure 2:**
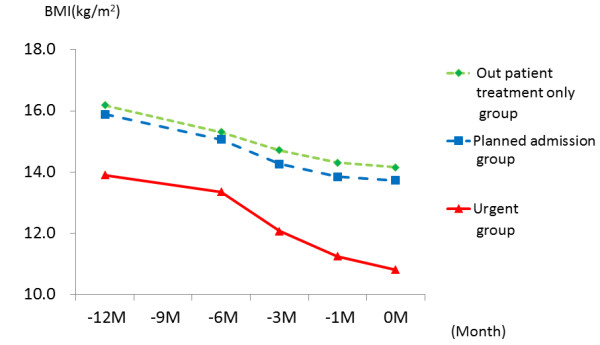
**Longitudinal pattern of BMI over the year before the first consultation**.

The changes in BMI over the previous year of the urgent (n = 17), planned admission (n = 96) and outpatient groups (n = 136) are shown in Figure 2. The BMI's over the past year (12,6,3,1,and 0 months before) of the urgent hospitalization group were 13.9 ± 2.5, 13.4 ± 1.9, 12.1 ± 1.2,11.3 ± 1.3, and 10.8 ± 1.4 kg/m^2^(mean ± SD), respectively. Those of the planned admission group were 15.9 ± 3.1, 15.1 ± 2.6, 14.3 ± 2.0,13.9 ± 1.7, and 13.7 ± 1.7 kg/m^2 ^respectively. Those of the outpatient treatment only group were 16.2 ± 2.8 15.3 ± 2.4, 14.7 ± 2.1,14.3 ± 1.9, and 14.2 ± 1.8 kg/m^2^, respectively. In the planned admission and outpatient groups, BMI followed a similar course along the temporal axis and values. Subsequently, we treated these groups as one (all control group) in study 1 with regard to physical severity.

Comparison of the urgent and all control groups found no difference in age (25.6 ± 9.7 vs., 22.5 ± 8.6 yr), type of AN, or sex (Table 1). The urgent group had a significantly longer duration of illness than the all control group (P < 0.05), and the BMI at first visit was significantly low (P < 0.01).

**Figure 3:**
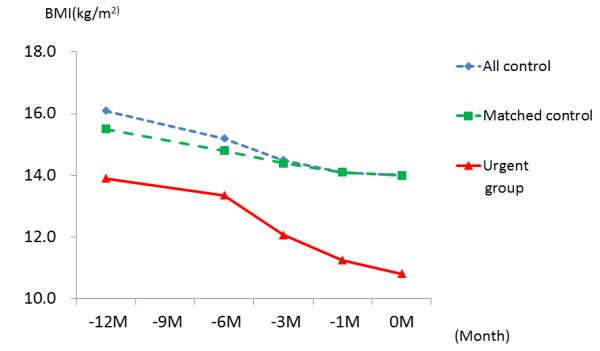
**Differences in longitudinal BMI pattern between the urgent hospitalization and the other AN groups**. All control: Planned admission group+ outpatient treatment only group Matched control: the duration of disease and age of the urgent group were matched with the all control group.

Differences in longitudinal BMI patterns of the urgent and matched control groups are shown in Figure 3.The BMI course of the urgent group was significantly different from that of the matched control group (P < 0.001). The BMI of the urgent group was already lower than that of the matched controls 12 months before, even when adjusted for the disease period and age (Table 2). Furthermore, the urgent group had rapidly decreasing weight as the patients got closer to admission, significantly decreased in comparison with the matched controls. In other words, a high interaction was found in the pattern of the time course towards admission and the BMI of both groups, even when adjusted for the disease period and age.

**Table 1:** Clinical characteristics of the urgent hospitalization and all control groups.

	Urgent group (n = 17)	All control (n = 232)	p
**Age (y)**	25.6 ± 9.7	22.5 ± 8.6	.13
**Sex (male/female)**	1/18	7/224	.52
**Duration (y)**	7.3 ± 7.4	4.5 ± 5.4*	.04
**Subtype(AN-R/AN-BP)**	9/8	119/112	.92
**BMI at first visit (kg/m^2^)**	10.8 ± 1.4	14.0 ± 1.8*	.00

All control: Outpatient treatment only group + planned admission group.

AN-R: Anorexia nervosa restricting type AN-BP: Anorexia nervosa binge eating and purging type * P < 0.05

**Table 2:** Association between BMI and urgent hospitalization by Repeated measures ANOVA for matched pairs.

Drive	df	F value	Pr>F
**Urgent hospitalization**	1	17.87	<0.0001
**Time**	2	31.46	<0.0001
**Urgent hospitalization** **X time**	2	11.36	<0.0001

BMI: body mass index ANOVA: analysis of variance df: degree of freedom

**Figure 4:**
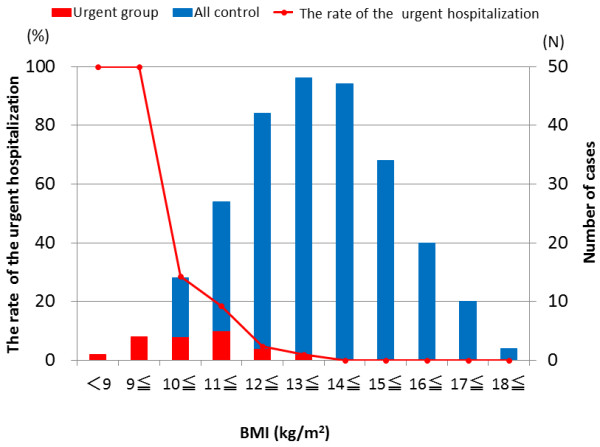
**The rate of urgent hospitalization, by BMI**. The bar graph shows the number of patients of each group. The line graph indicates the number of urgent hospitalizations by BMI. BMI: Body mass index.

The rates of urgent hospitalization for each BMI level on consultation day are shown in Figure 4. The rate of urgent hospitalization significantly rose with a decrease in BMI (P < 0.001). All of the patients required urgent hospitalization when BMI became less than 10 kg/m^2^.

### Study 2

Ten of the 17 patients of the urgent group and 75 of the 96 patients of the planned admission group were the subjects of this study.

**Figure 5:**
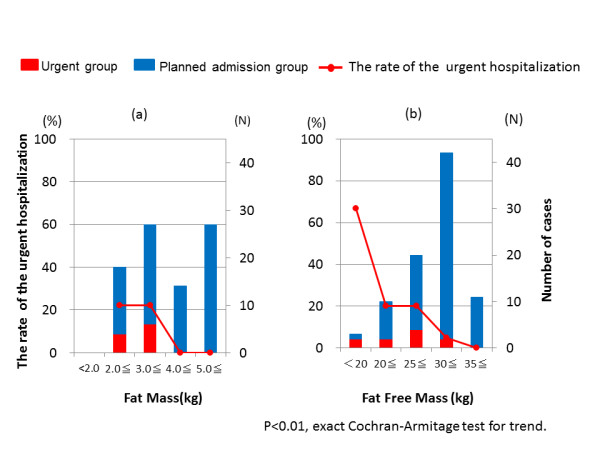
**The rate of urgent hospitalization, by FM and FFM**. The bar graph shows the number of patients of each group: FM (a) and FFM(b). The line graph indicates the number of urgent hospitalizations: FM(a) and FFM (b). FM; fat mass FFM: fat free mass.

The fat mass (FM) and fat free mass (FFM) of the urgent group were significantly lower than for the planned admission group (FM; 2.79 ± 0.48 vs. 4.76 ± 2.3 kg, P = 0.008, FFM; 25.2 ± 4.6 vs. 31.4 ± 4.4 kg, P = 0.00). Urgent hospitalization was required when FM was less than 3.6 kg and FFM less than 31.1 kg. The rate of urgent hospitalization rose with significantly decreased FFM and FM (P = 0.004 and P = 0.004, respectively Figures 5a, 5b). Urgent hospitalization gradually increased when FFM decreased to less than 35 kg. However, it remained at the same level when FM decreased to less than 4.0 kg.

In the comparison of the socio-demographic variables, significantly more in the urgent group were married than in the planned admission group (41.2% vs. 15.5%: P = 0.095). No other significant between group differences were found in other social support factors (Data not shown).

## Discussion

The BMI course and body composition of AN patients who developed disturbance of consciousness and/or difficulty walking were clearly different from those of the control group patients. Quantitative analysis showed that the urgent hospitalization group had a lower BMI one year before admission and that weight loss was significantly more rapid than that of the control group. The rate of urgent hospitalization increased with a reduction in BMI, FFM and FM. No considerable difference in social factors was found between the urgent and planned admission groups. It is not surprising clinically that patients at very low BMI require urgent hospitalization more often than those at higher BMI. We felt that it was important to empirically demonstrate this relationship and hope that our proof using the statistical procedure is useful for primary physicians in their efforts to better care for their AN patients.

For patients with AN, low BMI at referral indicates a substantial risk for chronic AN and death related to emaciation [12]. A BMI of less than 13 kg/m^2 ^has been proposed as a cutoff point for a poor prognosis [12]. Another paper showed that the relative risk of infection is 11.6 times greater for patients with BMI<12 kg/m^2 ^[13]. Recently, we reported BMI of 13-14 kg/m^2 ^to be the boundary at which body composition changes significantly and that BMI and FFM and FM had a curvilinear relation at the time of hospitalization[14]. In this study, FFM was more closely associated than FM with urgent hospitalization. It is interesting that FFM, which represents muscle and internal organ tissue, is more closely related to urgent hospitalization than FM, which represents energy storage. In addition, there were no cases of FM decreasing to 2.0 kg or less, even if the weight was extremely decreased. FM may play some kind of role as a life support.

When BMI is 13-14 m/kg^2 ^or more, the body uses fat as energy under conditions of starvation. When BMI is lower than 13-14 m/kg^2^, the supply of energy is converted from fat mass to protein. 1 g of fat mass (9 kcal/g) has about twice the calories of protein (4 kcal/g). It is speculated that the speed of BMI reduction increases with the same caloric output if BMI becomes low (<13-14 kg/m^2^). For the causes of disturbance of consciousness, hypoglycemia and abnormality of the cardiovascular and peripheral vascular systems are suggested[15]. For gait disturbance, reduction of muscle mass, abnormality of metabolic systems, including electrolyte imbalance and dehydration, are suggested[16]. The hypoglycemia of AN patients is generally mild, and it rarely reaches hypoglycemic coma [17]. It is suggested that it is counteracted by the secretion of counter hormones such as adrenocorticotropic hormone(ACTH) or growth hormone(GH)[17]. In a state of starvation, the muscle tissue becomes an important resource for glycogenesis. From these facts, the following are suggested. Values for BMI of 13 m/kg^2^, FFM of 30 kg, and FM of 3.0 kg may indicate the turning point of the failure of the homeostasis mechanism in the starvation state and be the stage before the development of a serious physical crisis.

BMI alone on the first consultation day is not sufficient to adequately determine the physical situation of AN patients. However, temperature or blood pressure used as guidelines for the necessity of urgent hospitalization fluctuate intensely along with changes in the physical state, making them difficult to use as a predictors. We suggest that the pattern of BMI (Figure 4) and FFM (Figure 5b) are more predictive and easy to use than the above mentioned factors, and their use will contribute not only to the welfare of patients but also to medical economy by preventing physically severe onset at the primary care stage. The definition of the urgent group was done strictly, as described in methods. It is possible that the data in study 2 would have been more accurate if the data of 16 hospitalized participants who were placed in the planned admission group were instead added to the urgent group.

Most social background factors showed no between group differences. Although age was not different, the percentage who were married was high in the urgent hospitalization group. The above suggests that the decision as to the need for urgent hospitalization was made mainly by physical factors, with very little contribution of factors related to social background.

From the aspects of psychosocial and genetic background, we cannot determine the factors responsible for the differences between the urgent admission and control groups in this study. There is undoubtedly a genetic predisposition and a range of environmental risk factors in the pathogenesis of eating disorders [1,18]. Numerous factors related to the serious physical state of AN patients are included in their psychological evaluation, and the patient's psychological status tends to fluctuate in the early period of treatment. These factors are too complex to evaluate in this paper, so we have left them for future study. Virtually nothing is known about the individual causal processes involved or about how they interact and vary across the development and maintenance of the disorders[18]. It is known that starvation shrinks the brain and is associated with many psychological disturbances, such as rigidity, emotional deregulation, and social difficulties [1]. This vicious cycle might develop gradually into a weight decrease.

Limitations: This study applies data on patients who visited a hospital; thus, data on AN sufferers who did not visit a hospital are not included. Only hospitalized patients were included in the study of body composition. There was no change in the BMI before hospitalization between the outpatient and hospitalized groups. Therefore we substituted perspective by an evaluation of the hospitalized group in this assessment. There is a risk when using the data from a single institution. Comorbidity is the rule rather than the exception for patients with eating disorders[1]. The social background factors that were evaluated may not be complete. It will be necessary to consider the income of the family and the convenience of the access to the hospital in future study. It is important that we evaluate certain personality traits such as perfectionism, obsessive-compulsive tendencies, social withdrawal, and depression. An evaluation of comorbidites and the psychological severity are future themes.

In conclusion, the pattern of BMI change and FFM may be useful for understanding the physical severity in AN. More research is needed on the accurate prediction of a need for urgent hospitalization. However, this combination of BMI and FFM might be useful for clinicians to monitor to help them avoid urgent hospitalization of their AN patients.

## Competing interests

The authors declare that they have no competing interests.

## Authors' contributions

KK designed the study, collected the data, analysed the data, interpreted the results, and drafted the manuscript. SY participated in the design of the study, interpret the results and reviewed the manuscript. TY participated in the design of the study and performed the statistical analysis. MG, CM, TN, ST, TH,YY and MT helped the collection of data and interpret the results. SM, CK and NS participated in the coordination of the study and reviewed the manuscript. All authors read and approved the final manuscript.
